# Antibodies against Human Cytomegalovirus in the Pathogenesis of Systemic Sclerosis: A Gene Array Approach

**DOI:** 10.1371/journal.pmed.0030002

**Published:** 2005-12-06

**Authors:** Claudio Lunardi, Marzia Dolcino, Dimitri Peterlana, Caterina Bason, Riccardo Navone, Nicola Tamassia, Ruggero Beri, Roberto Corrocher, Antonio Puccetti

**Affiliations:** **1**Department of Clinical and Experimental Medicine, Section of Internal Medicine, University of Verona, Verona, Italy; **2**Institute Giannina Gaslini, Genova, Italy; **3**Department of Pathology, Section of General Pathology, University of Verona, Verona, Italy; **4**Department of Experimental Medicine, Section of Histology, University of Genova, Genova, Italy; Leiden University Medical CenterNetherlands

## Abstract

**Background:**

Systemic sclerosis is an autoimmune disease characterized by immunological abnormalities, vascular damage, and fibroblast proliferation. We have previously shown that a molecular mimicry mechanism links antibodies against the human-cytomegalovirus-derived protein UL94 to the pathogenesis of systemic sclerosis. The UL94 epitope shows homology with NAG-2, a surface molecule highly expressed on endothelial cells. Anti-UL94 peptide antibodies purified from patients' sera induce apoptosis of endothelial cells upon engagement of the NAG-2–integrin complex.

**Methods and Findings:**

We show here that NAG-2 is expressed on dermal fibroblasts and that anti-UL94 antibodies bind to fibroblasts. We have used the gene array strategy (Affimetrix oligonucleotide microarrays) to analyze the transcriptional profile in response to a 4-h and an 8-h treatment with antibodies against the UL94 peptide in endothelial cells and dermal fibroblasts. Exposure of endothelial cells to anti-UL94 antibodies had a profound impact on gene expression, resulting in the upregulation of 1,645 transcripts. Several gene clusters were upregulated including genes encoding adhesion molecules, chemokines, colony-stimulating factors (CSFs), growth factors, and molecules involved in apoptosis. Following antibody stimulation, dermal fibroblasts showed an upregulation of 989 transcripts and acquired a “scleroderma-like” phenotype. Indeed, genes involved in extracellular matrix deposition, growth factors, chemokines, and cytokines were upregulated. We confirmed the microarray results by real-time quantitative polymerase chain reaction and by measuring some of the corresponding proteins with ELISA and Western blotting.

**Conclusion:**

Our results show that anti-human-cytomegalovirus antibodies may be linked to the pathogenesis of systemic sclerosis not only by inducing endothelial cell activation and apoptosis but also by causing activation of fibroblasts, one of the hallmarks of the disease.

## Abstract 2

Anti-human-cytomegalovirus antibodies may be linked to the pathogenesis of systemic sclerosis by inducing endothelial cell activation and apoptosis, and fibroblast activation and proliferation.

## Introduction

Systemic sclerosis (SSc) is an autoimmune disease characterized by three main features: (i) structural and functional vascular abnormalities with perivascular infiltration of mononuclear inflammatory cells, intimal proliferation, and luminal narrowing at both the arteriolar and arterial levels, (ii) immunologic abnormalities, both humoral and cellular, such as the presence of autoantibodies to intracellular and cell surface antigens, and perivascular T cell infiltration of the skin and internal organs, and (iii) excessive extracellular matrix deposition, leading to fibrosis of the skin and of internal organs [1].

Autoantibodies directed against intracellular antigens are associated with SSc and differentiate two distinct clinical subsets: anticentromere antibodies are found in SSc with limited cutaneous involvement, while anti–DNA topoisomerase I antibodies are associated with SSc with diffuse cutaneous involvement [2]. Moreover, autoantibodies directed against cell surface antigens might induce endothelial cell damage and apoptosis, considered a primary event in the pathogenesis of the disease [3,4].

Latent human cytomegalovirus (hCMV) infection may contribute to progression of SSc through its ability to infect endothelial cells [5]. Indirect evidence for the association between hCMV and SSc comes from the prevalence of anti-hCMV antibodies in patients affected by the disease [6]. Moreover, monoclonal antibodies against topoisomerase I were found to recognize a pentapeptide of the autoantigen sharing homology with the hCMV-derived UL70 protein, suggesting the activation of autoreactive B cell clones by a molecular mimicry mechanism [7]. Furthermore, some patients with chronic graft-versus-host disease develop SSc-like lesions with the presence of typical autoantibodies such as anti–topoisomerase I [5], and hCMV infection is associated with an increased risk for the development of chronic graft-versus-host disease [8]. Finally, murine sclerodermatous graft-versus-host disease is one of the animal models for human scleroderma [9,10].

In a previous study we provided direct evidence for a molecular mimicry mechanism by which antibodies against a hCMV-derived protein can be linked to endothelial cell damage in patients with SSc [11]. In the majority of patients' sera there are antibodies directed against an epitope (VTL GGAGIWLPP) contained within UL94, a hCMV-derived protein expressed in infected cells with very late kinetics. UL94 is localized in the nucleus of infected cells and may be involved in the regulation of viral and/or cellular gene expression. The UL94 epitope shows homology with NAG-2 [12], a cell surface molecule highly expressed on non-stressed endothelial cells and associated with integrins. Affinity purified anti-UL94 peptide IgG antibodies recognize NAG-2 in a whole cell lysate and induce apoptosis of non-stressed endothelial cells upon engagement of the NAG-2–integrin complex [11]. Therefore, we propose that hCMV is linked to the pathogenesis of SSc through a particular subset of anti-hCMV antibodies that specifically interacts with a normally expressed endothelial cell surface receptor sharing similarity with the UL94 viral protein. The engagement of the receptor results in endothelial cell apoptosis, considered the primary pathogenic event in SSc.

Another fundamental feature of SSc is the fibrosis of the skin and internal organs because of increased extracellular matrix deposition [13]. Indeed, fibroblasts are thought to play a major role in the pathogenesis of the disease. They are directly involved in the synthesis of many extracelluar matrix components, and the dysregulation of extracellular matrix turnover is central to fibrosis development in SSc. Scleroderma fibroblasts display a variety of phenotypic defects that range from increased synthesis of multiple matrix proteins to abnormalities of cell surface receptors and signaling pathways [14].

While a direct link between endothelial cell damage in SSc and hCMV infection has been shown, a correlation between hCMV and fibrosis is still lacking. In the present study we wanted to verify whether the NAG-2 receptor is expressed also on normal fibroblasts and whether the anti-hCMV antibodies bind normal dermal fibroblasts upon interaction with the NAG-2 receptor. Moreover, we decided to use a DNA microarray approach to analyze the effects of these antibodies on endothelial cells and fibroblasts, in order to better understand the role played by hCMV in the pathogenesis of the disease. Our findings at gene level were confirmed with real-time quantitative polymerase chain reaction and with the analysis of a panel of proteins both in the supernatants of the cells exposed to the antibodies and in the sera of patients affected by SSc.

## Methods

### Patients and Controls

Eighty-one SSc patients (75 women and six men, mean ± standard deviation age 54.5 ± 14.1 y) from northern Italy were studied. All patients fulfilled the American College of Rheumatology criteria for SSc [15]. Sixty of them were affected by limited cutaneous SSc and 21 by diffuse cutaneous SSc. Sixty sex- and age-matched individuals without SSc were used as controls. Blood was obtained from all the patients and controls after informed written or oral consent.

### Peptide Synthesis

The UL94-derived peptide (VTL GGAGIWLLP) and the irrelevant control peptide (VTLPKDSDVELP)—chosen on the basis of its absence of relevant homology with the viral peptide, with the NAG-2 molecule, and with human self-antigens according to BLASTP via the NCBI BLAST network service [11]—were synthesized by solid phase synthesis with the 9-fluorenylmethoxycarbonyl strategy [16].

### Purification of Anti-Peptide Antibodies

To affinity purify IgG antibodies against either the UL94 peptide or the control peptide, each peptide (5 mg/g of dried sepharose powder) was coupled to sepharose 4B (Pharmacia, Uppsala, Sweden), according to the manufacturer's instructions. Pooled sera from ten different patients with SSc were diluted in PBS. Aliquots of the diluted serum samples were applied to each column separately. The columns were washed with PBS. Bound immunoglobulins were eluted with 0.1 M glycine (pH 2.5) and dialyzed against PBS.

### Cell Culture

Human endothelial cells (HUVECs) and human dermal fibroblast as well as their growth media were purchased from Promocell Bioscience Alive (Heidelberg, Germany). HUVECs were used between passages 2 and 5, while dermal fibroblasts between passages 3 and 6. Cell monolayers were stimulated with antibodies affinity purified against the UL94 peptide or the irrelevant control peptide (20 μg/ml) for various intervals of time as described in the text. Cell pellets were used for gene array experiments, while soluble mediators were measured in the cell supernatants.

### FACS Analysis

For FACS (fluorescence-activated cell sorting) analysis, cells were incubated with specific or control antibodies (5 μg/ml) for 30 min on ice. Antibody binding was revealed with fluorescein-isothiocyanate-conjugated anti-human IgG antibodies (SouthernBiotech, Birmingham, Alabama, United States). Samples were run on a FACScan flow cytometer (Becton Dickinson, Franklin Lakes, New Jersey, United States).

### Western Blot Analysis

Human dermal fibroblasts were lysed in cold lysis buffer (0.5% NP-40, 10 mM TRIS [pH 7.4], 0.15 M sodium chloride, 5 mM magnesium chloride), and lysates were immunoprecipitated with rabbit anti-NAG-2 antibodies [11] cross-linked to sepharose 4B (Pharmacia). Eluted proteins were resolved by SDS-PAGE and transferred to nitrocellulose membrane (Amersham Bioscience, Piscataway, New Jersey, United States). Blots were incubated with either anti-NAG-2 antibodies or human affinity purified anti-peptide antibodies (10 μg/ml). The Renaissance Chemiluminescence Kit (NEN, Boston, Massachusetts, United States) was used for detection.

### Competitive Enzyme-Linked Immunosorbent Assay

Enzyme-linked immunosorbent assays (ELISAs) were carried out as previously described [11,17]. Briefly, for the binding of antibodies to fibroblasts, affinity purified anti-hCMV peptide antibodies (10 μg/ml) were diluted in PBS containing 1% BSA and were incubated for 1 h at 37 °C with fibroblasts that had been previously fixed with 0.1% glutaraldehyde. Bound IgG was detected by a further 1 h incubation with a peroxidase-conjugated antibody against human IgG (Amersham Bioscience). For the competitive assay, the amount of antibody that gave 50% of the maximum binding to fibroblast was preincubated for 1 h at 37 °C with different amounts of competitors or buffer and then transferred to the fibroblast-coated plates. The assay was then carried out as the direct binding assay.

### Proliferation Assay

To assess cell proliferation, fibroblasts (5000 cells/well) were cultured for various intervals of time in microtiter plates in the presence or absence of antibodies (15 μg/ml affinity purified antibodies). Cell viability was assessed using the commercially available kit (Alexis Biochemicals, San Diego, California, United States).

### Preparation of cRNA and Array Analysis

Preparation of cRNA, hybridization, and scanning of probe arrays were performed according to the protocols of the manufacturer (Affymetrix, Santa Clara, California, United States) by the Genopolis Consortium (University of Milano-Bicocca, Italy) using the Human Genome U133A GeneChip (Affymetrix). The Human Genome U133A GeneChip is a single array representing 14,500 well-characterized human genes and including more than 22,000 probe sets and 500,000 distinct oligonucleotide features.

The different gene expression patterns were analyzed by using Array Assist version 2.0 (Stratagene, La Jolla, California, United States), which calculated a robust multi-array average of background-adjusted, normalized, and log-transformed intensity values applying the robust multi-array average algorithm [18,19]. With this software, the mean optical background level for each array was subtracted from the signal intensity for each probe. Finally, the normalized, background-corrected data were transformed to the log_2_ scale. A signal log_2_ ratio of 1.0 indicates an increase of the transcript level by 2-fold change (2 F.C.) and −1.0 indicates a decrease by 2-fold (−2 F.C.). A signal log_2_ ratio of zero would indicate no change.

In our study, we analyzed the gene expression profiles in both endothelial cells and fibroblasts stimulated with antibodies affinity purified against the UL94 peptide (test samples) or with antibodies affinity purified against an irrelevant peptide (control samples) for 4 and 8 h. Genes were selected for final consideration when their expression (F.C.) was at least 2-fold different in the test sample versus control sample at at least one time point. The microarray results have been reported according to the MIAME guidelines and deposited in the public repository ArrayExpress (http://www.ebi.ac.uk/arrayexpress; accession number E-MEXP-382).

### Quantitative Real-Time Polymerase Chain Reaction

Total RNA was extracted from endothelial cells and fibroblasts using TRIzol reagent (Invitrogen, Carlsbad, California, United States), following manufacturer's instructions. One microgram of total RNA from each sample was treated with amplification grade DNase I and then used as a template for the reverse transcription reaction, using random hexamers and SuperScript II Reverse Transcriptase (Invitrogen). All samples were reverse transcribed under the same conditions and from the same reverse transcription master mix, in order to minimize differences in reverse transcription efficiency. Triplicate quantitative real-time polymerase chain reactions (Q-PCRs) for each sample were performed in 20 μl containing 20 ng of cDNA, 10 μl of 2× Platinum SYBR Green qPCR SuperMix UDG (Invitrogen), and primers at a final concentration of 200 nM. Table 1 shows the primers used. The Q-PCR reactions were performed in 96-well plates with optical caps using the DNA Engine Opticon 2 System (MJ Research, Waltham, Massachusetts, United States). The reaction conditions were identical for all primer sets as follows: 50 °C for 2 min, 95 °C for 2 min, and then 40 cycles of 95 °C for 15 s and 60 °C for 1 min. Control wells containing no template were used to exclude the presence of contaminating template molecules and to identify potential primer–dimer products from the dissociation curve analysis. Amplification plots were analyzed using Opticon Monitor version 2.02 (MJ Research), and data were calculated with QGene (http://www.qgene.org/) and expressed as mean normalized expression. *Glyceraldehyde-3-phosphate dehydrogenase (GAPDH)* was selected as a normalizing gene according to its stable expression levels.

**Table 1:**
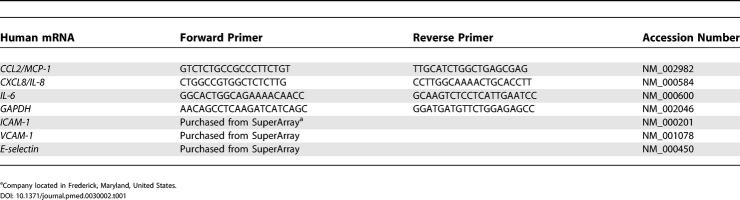
Primers Used for Q-PCR

### Cytokines, Chemokines, and Adhesion Molecules

Aliquots of sera and supernatants were frozen at −80 °C until assayed. The soluble mediators were measured with commercially available ELISAs according to the manufacturer's protocol. Measurement below detectable levels was used as the lower cut-off limit of the assay, according to the instructions of the manufacturer. The value recorded was the mean of two measurements. ELISA kits for Vascular endothelial growth factor (VEGF), Interleukin-6 (IL-6), IL-8, Transforming growth factor beta 1 (TGF-beta 1), and Monocyte chemotactic protein 1 (MCP-1) were purchased from Amersham Bioscience; IL-11, MCP-3, soluble Intercellular adhesion molecule 1 (ICAM-1), soluble E-selectin, and Vascular cell adhesion molecule 1 (VCAM-1) from R&D Systems (Minneapolis, Minnesota, United States); Interferon-gamma-inducible protein 10 (IP10) from Bender MedSystems (Vienna, Austria); Endothelin 1 (ET-1) from Assay Designs (Ann Arbor, Michigan, United States); C-terminal Propeptide of Collagen type I (Pro-Col I) from Quidel Corporation (San Diego, California, United States); and Epidermal growth factor (EGF) from Chemicon International (Temecula, California, United States).

### Gene Ontology Analysis

We performed a Gene Ontology (GO) analysis using Array Assist version 2.0 (Stratagene).

### Statistical Analysis

Statistical testing was performed using StatsDirect (StatsDirect, Cheshire, United Kingdom). The significance of differences between patients and controls was determined using the unpaired Student's *t*-test; *p* < 0.05 was considered statistically significant. For sake of clearness the values are expressed as mean with 95% confidence interval.

## Results

### Anti-hCMV Peptide Antibodies Bind to Normal Dermal Fibroblasts

**Figure 1:**
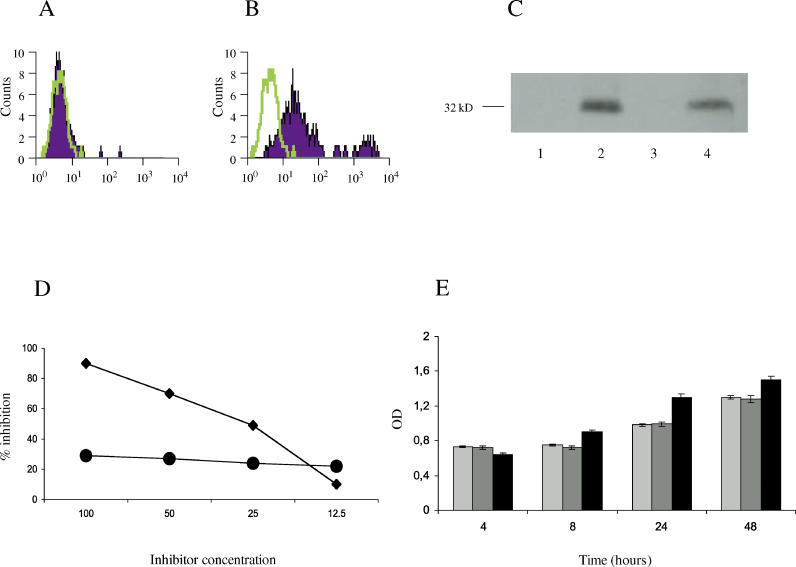
Anti-hCMV Antibodies Recognize the NAG-2 Receptor on Dermal Fibroblasts and Do Not Affect Cell Proliferation (A and B) FACS analysis of the binding of antibodies to normal human fibroblasts: antibodies affinity purified against the irrelevant peptide from sera of SSc patients (A) and antibodies affinity purified against the UL94 peptide from the same sera (B). Percentage of positive cells is 7% (A) and 83 % (B), respectively. (C) Anti-peptide antibodies react with the NAG-2 molecule. Lysates from human dermal fibroblasts were immunoprecipitated with a rabbit affinity purified anti-UL94 peptide antibody crosslinked to sepharose. Immunoprecipitates were resolved in SDS-PAGE and transferred to nitrocellullose. Blots were incubated with pre-immune rabbit antiserum (lane 1), rabbit anti-UL94 peptide antibody (lane 2), affinity purified antibodies directed against the irrelevant control peptide isolated from patients with SSc (lane 3), and anti-UL94 peptide antibodies purified from the sera of the same patients (lane 4). (D) Binding of affinity purified antibodies against the UL94 peptide to fibroblasts is specifically inhibited by the UL94 peptide (diamonds) but not by an irrelevant control peptide (circles). Data represent percent of inhibition; inhibitor concentration (horizontal axis) is in micrograms per milliliter. (E) Fibroblasts incubated with anti-hCMV antibodies normally proliferate. Shown are proliferation levels for fibroblasts incubated for various intervals of time with medium alone (light grey bars), with affinity purified antibodies directed against the irrelevant control peptide (dark grey bars), or with affinity purified antibodies against the UL94 peptide (black bars). OD, optical density at 570 nm.

To verify whether anti-hCMV peptide antibodies bind to human dermal fibroblasts, we performed a FACS analysis using affinity purified anti-UL94 peptide IgG antibodies and dermal fibroblasts. As shown in Figure1A and 1B, anti-peptide antibodies were able to bind dermal fibroblasts. We also showed that the NAG-2 receptor is expressed on the surface of dermal fibroblasts and that this molecule is recognized by anti-hCMV peptide antibodies (Figure 1C). The specificity of the interaction of such antibodies with the NAG-2 receptor was further confirmed by a competitive ELISA that demonstrated that the viral peptide could displace the binding of the antibodies from the surface of the dermal fibroblasts (Figure 1D). Since we have previously demonstrated that anti-hCMV antibodies are able to inhibit cell proliferation and induce apoptosis of endothelial cells upon engagement of the NAG-2 molecule [11], we next wanted to evaluate whether the interaction between the antibodies and dermal fibroblasts had some functional consequence for these cells. As shown in Figure 1E, dermal fibroblasts incubated with the anti-hCMV antibodies proliferated normally, and after 24 and 48 h the rate of proliferation was slightly higher than for cells incubated with antibodies against an irrelevant peptide. These data indicate that anti-hCMV peptide antibodies recognize NAG-2 expressed on dermal fibroblasts and that this interaction does not inhibit cell proliferation of the target cells.

### Gene Expression Profile in HUVECs Treated with Anti-hCMV Peptide Antibodies

Since we have already shown that anti-UL94 peptide antibodies promote endothelial cell apoptosis following engagement of the NAG-2 molecule [11], we decided to analyze the gene expression profiles induced in endothelial cells by the anti-hCMV antibodies in order to identify clusters of genes known to be involved in the pathogenesis of vascular damage in SSc.

For this purpose normal endothelial cells were incubated with either anti-UL94 peptide antibodies affinity purified from the sera of patients with SSc or with control antibodies affinity purified against an irrelevant peptide from the sera of the same individuals. The gene expression profiles were studied at two different time points: after 4 and 8 h of stimulation. As stated in Methods, we considered only those genes expressed more than 2-fold above control at minimally one time point. Using these criteria, anti-hCMV antibodies were found to upregulate 1,645 transcripts (Dataset S1) including genes encoding adhesion molecules, chemokines, CSFs, growth factors, and molecules involved in apoptosis.

**Figure 2:**
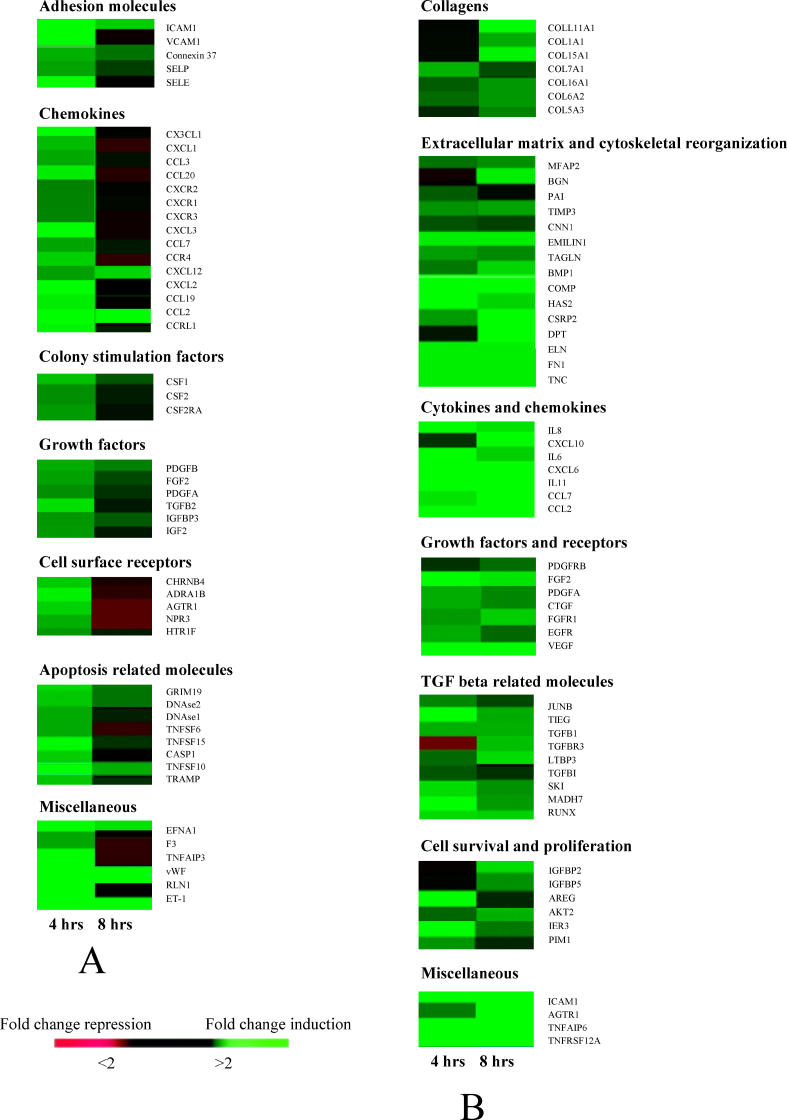
Profile of Selected Genes Modulated in Response to Anti-hCMV Antibodies Figure shows F.C. in gene expression in endothelial cells (A) and fibroblasts (B) at 4 and 8 h after incubation with affinity purified anti-UL94 antibodies. Genes are grouped into functional categories based on our current understanding of their function. Genes shown have at least a 2-fold change in gene expression.

Figure 2 shows an overview of some genes within the above mentioned clusters. A more detailed representation of the same genes is presented in compiled form in Table 2, which includes GeneBank accession numbers and F.C. of expression of the genes. Among the genes encoding adhesion molecules, the highest increase in expression was observed for E-selectin, VCAM-1, and ICAM-1 coding genes (F.C. 68.5, 26.5, and 18.8, respectively, at 4 h of stimulation) (Table 2). High circulating levels of these adhesion molecules have been found in scleroderma [20].

**Table 2:**
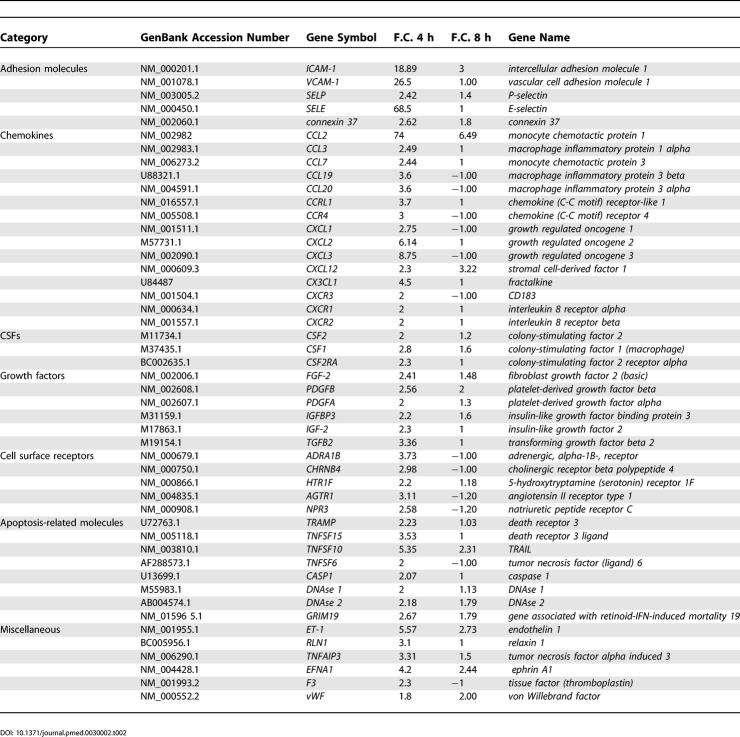
Gene Expression in HUVECs after 4 and 8 h of Antibody Stimulation

Another cluster of upregulated genes was represented by genes encoding chemokines (Table 2), a group of cytokines able to attract leukocytes and regulate angiogenesis, vascular proliferation, and fibrosis, functions that may contribute to the manifestations of scleroderma [21]. The expression of MCP-1 (also called CCL2) was highly increased, with a peak of 74-fold increase in expression at 4 h of stimulation. MCP-1 stimulates fibroblast proliferation and collagen production [22]. Several other chemokine genes were upregulated in endothelial cells treated with anti-hCMV peptide antibodies, such as those encoding MCP-3 (or CCL7), Macrophage inflammatory protein 1 alpha (MIP-1 alpha [or CCL3]), Growth regulated oncogene 1 (GRO 1 [or CXCL1]), Stromal cell-derived factor 1 (SDF-1), and Fractalkine.

Consistent with endothelial damage and activation, an increased expression of the genes encoding von Willebrand factor (vWF), Tissue factor (F3), Ephrin A1, and ET-1 was present in stimulated endothelial cells. Although the increase in expression of these genes is not specific for SSc, vWF and F3 molecules are associated with the acquisition of a procoagulant phenotype typical of activated endothelial cells and Ephrin A1 and ET-1 molecules are associated with diseases characterized by endothelial dysfunction [23]. Of note is the upregulation of the gene encoding Angiotensin II receptor type 1, which plays an important role in ischemia-induced angiogenesis [24].

Moreover, we found an increased expression of several genes encoding growth factors (Table 2), including CSFs, Fibroblast growth factor 2 (FGF-2), Platelet-derived growth factor (PDGF), and Insulin-like growth factor 2 (IGF-2). These molecules have also been implicated in the pathogenesis of SSc; for example, PDGF seems to be associated with fibrotic damage in SSc [25].

Finally, genes encoding molecules involved in the apoptotic process, such as TRAMP (DR3), GRIM19, Caspase 1, and DNAse 1 and 2, were upregulated in accordance with the observation that endothelial cells undergo cell death following incubation with anti-hCMV antibodies [11].

Taken together, these results show that a subset of anti-hCMV antibodies modulate the expression of a series of gene clusters encoding molecules playing a fundamental role in the vascular damage typical of SSc.

### Gene Expression Profile in Human Dermal Fibroblasts Treated with Anti-hCMV Peptide Antibodies

Human dermal fibroblasts were incubated with either anti-UL94 peptide antibodies affinity purified from the sera of patients with SSc or with control antibodies purified against an irrelevant peptide from the sera of the same individuals. The gene expression profiles were studied at the same time points as for endothelial cells, and we considered for further analysis only those genes whose expression increased more than 2-fold above control at at least one time point.

The engagement of the NAG-2 receptor by anti-hCMV antibodies upregulated 989 transcripts (Dataset S2), including genes encoding molecules involved in extracellular matrix deposition, growth factors, chemokines, and cytokines. Figure 2A shows an overview of a panel of genes within the above mentioned clusters. A more detailed representation of the same genes is presented in compiled form in Table 3. An excessive deposition of collagen and extracellular matrix is typical of scleroderma fibroblasts and leads to fibrosis of the skin and internal organs [13]. When compared to control cells, treated fibroblasts showed increased expression of genes encoding different types of collagens such as Collagen type I, type XVI, and type XI (Table 3). Similarly several other genes involved in extracellular matrix deposition were upregulated including those encoding Fibronectin, Emilin 1, Dermatopontin, Biglycan, Cartilage oligomeric matrix protein, and Tenascin C with F.C. in expression ranging from 3.53 to 6.82. Increased levels of the above mentioned proteins have been associated with SSc [26]. Also, the gene encoding Bone morphogenetic protein 1 (Procollagen C-proteinase), an enzyme important for the formation of mature collagens [27], was upregulated, possibly because of the elevated rate of collagen synthesis observed in treated fibroblasts. A very high level of induction was observed for the Hyaluronan synthase 2 gene, with a F.C. in expression of 18.12 at 4 h, which is compatible with the function of the enzyme in extracellular matrix metabolism [28].

**Table 3:**
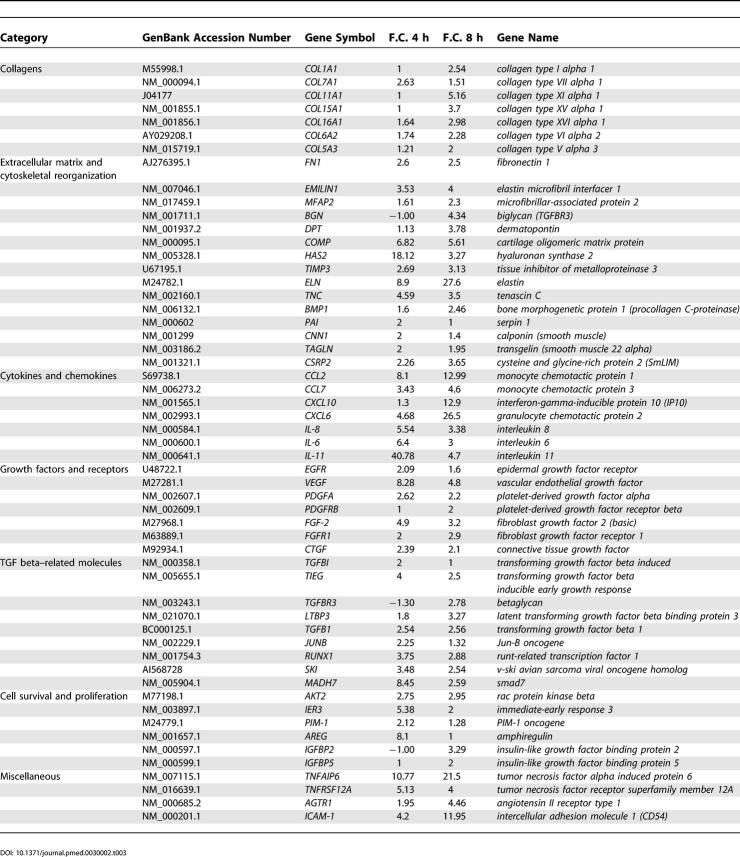
Genes Expression in Fibroblasts after 4 and 8 h of Antibody Stimulation

Analysis of gene expression patterns revealed that the fibroblasts used in this study also expressed some cytoskeletal genes encoding proteins typically associated with the myofibroblast phenotype (e.g., Transgelin and Elastin) [29]. Anti-hCMV antibodies also induced the expression of a number of cytoskeletal genes, which are usually expressed by highly differentiated smooth muscle cells, such as the gene for Calponin [29]. Of interest, we also observed induction of the smooth-muscle-cell-restricted signaling molecule Cysteine and glycine-rich protein 2 (CSRP2, also known as SmLIM for smooth muscle LIM-containing protein), which is expressed in differentiated vascular smooth muscle cells [30].

Overexpression of genes coding for proinflammatory and profibrotic cytokines (Table 3) has been observed: IL-6, IL-8, and IL-11, with F.C. in expression of 6.4, 5.54, and 40.78, respectively, at 4 h. Similarly, genes encoding some chemokines (see Table 2) were upregulated, including MCP-1, MCP-3, and IP10 with F.C. in expression of 12.9, 4.6, and 12.9, respectively, at 8 h of stimulation.

Another group of genes included those encoding growth factors typical of activated fibroblasts, among them TGF-beta 1 and its accessory receptor Betaglycan (TGFBR3) [31], and Connective tissue growth factor (CTGF), which represent key mediators of fibrosis in SSc [32]. Interestingly, a series of genes known to be upregulated in TGF-beta 1–treated normal fibroblasts [29,33] were found overexpressed in dermal fibroblasts exposed to anti-hCMV antibodies. This cluster of genes included those encoding the transcription factor JUNB, the Smad co-activator Runt-related transcription factor 1 (RUNX1), and the transcriptional regulator TIEG. Most importantly, we found an increased expression of the signaling molecule Smad7 (F.C. in expression of 8.45 at 4 h of stimulation), known to be overexpressed in scleroderma fibroblasts [34]. Also, the upregulation of Angiotensin II receptor type 1 is likely to significantly amplify the profibrotic actions of TGF-beta 1 [35]. Moreover genes coding for VEGF, PDGFA, and PDGF receptor B were overexpressed in treated fibroblasts.

Finally, we observed an increased expression of the gene coding for Rac protein kinase-beta (Akt), an important regulator of cell proliferation and survival, and, interestingly, this gene is overexpressed in scleroderma fibroblasts [36]. The induction of Akt along with that of two genes involved in regulating cell growth and apoptosis, IER3 [37] and PIM-1 [38], is consistent with the observation that anti-hCMV antibodies promote fibroblast survival.

Taken together, these results showed that genes involved in synthesis of extracellular matrix components and in cell survival and proliferation were upregulated in fibroblasts exposed to anti-hCMV antibodies.

### Downregulated Genes in Endothelial Cells and Fibroblasts

The engagement of the NAG-2 receptor by anti-hCMV antibodies downregulated 1,389 genes in endothelial cells and 931 genes in fibroblasts (Datasets S3 and S4). We selected a few of them, based on their functional relevance.

Table 4 shows a list of repressed genes in endothelial cells. The gene encoding the anti-apoptotic molecule BCL2 [39] was highly repressed, in keeping with the observation that endothelial cells undergo apoptosis following engagement of the NAG-2 receptor. The decreased expression of the gene encoding Endothelial nitric oxide synthase (eNOS) has already been reported in endothelial cells isolated from patients with SSc, indicating an intrinsic defect in the mechanism of nitric oxide production [40]. It is worth noting the downregulation of the Endothelin type B receptor since this receptor on endothelial cells promotes vasodilatation through release of nitric oxide and prostacyclin, increases the clearance of ET-1, and inhibits Endothelin-converting enzyme expression [41].

**Table 4:**
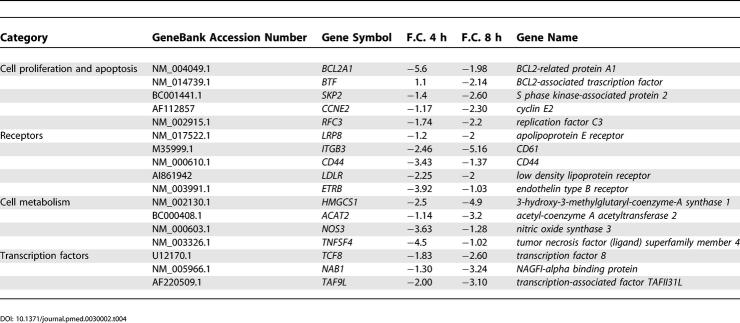
Downregulated Genes in HUVECs after 4 and 8 h of Antibody Stimulation

Table 5 summarizes the downregulated genes in fibroblasts. Interestingly the Death-associated protein kinase 1 (DAPK-1), a pro-apoptotic protein, was reduced in fibroblasts with a F.C. in expression of −4.47 and −7.5 at 4 and 8 h, respectively [42]. Also genes encoding matrix metalloproteinase proteins (MMP-1, MMP-3, and MMP-10) were reduced especially at 8 h after stimulation. These enzymes are responsible for matrix degradation and turnover and therefore their reduction may contribute to the pathogenesis of fibrosis [43,44]. Among the signal transduction molecules, Smad3, Smad1, and TGF-beta receptor 2 were downregulated.

**Table 5:**
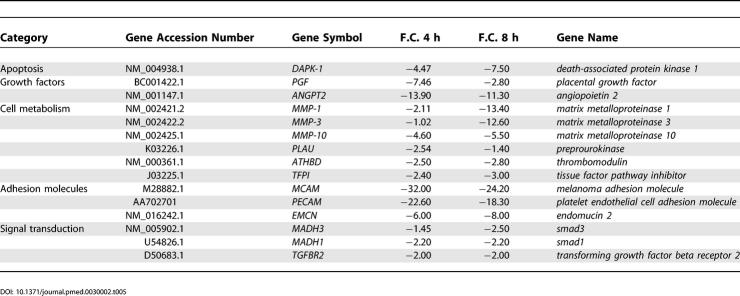
Downregulated Genes in Fibroblasts after 4 and 8 h of Antibody Stimulation

### Q-PCR Validation of Microarray Results

**Figure 3:**
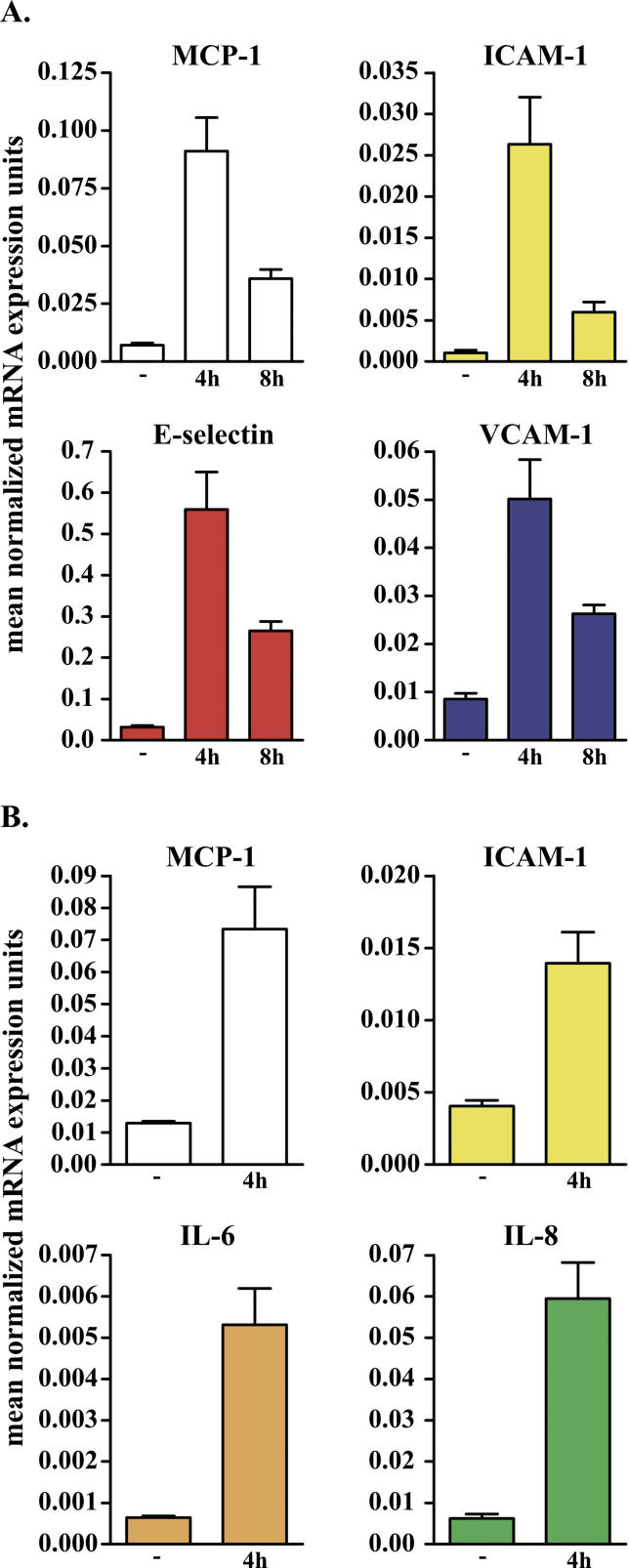
Validation of Gene Array Results by Q-PCR Genes selected for validation by Q-PCR in endothelial cells (A) and fibroblasts (B). MCP-1, ICAM-1, E-selectin, and VCAM-1 transcripts were increased by 12.89-, 25.36-, 17.59-, and 5.86-fold, respectively, in endothelial cells incubated with the antibodies for 4 h compared with control endothelial cells (A). MCP-1, ICAM-1, IL-6, and IL-8 transcripts were increased 5.7-, 3.44-, 9.54-, and 8.22-fold in fibroblasts incubated with the antibodies compared to control fibroblasts (B). The level of transcript expression is reported on the vertical axis. *GAPDH* was selected as endogenous gene.

We performed Q-PCR using the same endothelial and fibroblast total RNA as was used for the microarray experiments (Figure 3). Overall, the Q-PCR results were concordant with the array results in six of six genes tested, in terms of significant differences in expression between cells incubated with anti-UL94 antibodies and control samples. The genes subjected to validation included those encoding MCP-1, ICAM-1, E-selectin, and VCAM-1 in endothelial cells at 4 and 8 h (Figure 3A) and the genes encoding MCP-1, ICAM-1, IL-6, and IL-8 in fibroblasts at 4 h (Figure 3B).

*GAPDH* was selected as endogenous standard, and we saw no significant changes in the Q-PCR results when the data were normalized using *beta-actin,* another constitutively transcribed gene.

### Validation of Genes Induced by Anti-hCMV Peptide Antibodies via Analysis of Soluble Molecules Released in Cell Supernatants

The analyses of gene expression profiles and Q-PCR were paralleled by the analysis of some of the corresponding soluble mediators released by endothelial cells and fibroblasts. Table 6 shows the concentration of the molecules tested in the supernatants of cells incubated with antibodies against the irrelevant peptide and with anti-UL94 peptide antibodies. Endothelial cell supernatants were evaluated until the 24th hour of incubation, at which point apoptosis is nearly complete. In the supernatant of endothelial cells an increased concentration of the two chemokines MCP-1 and MCP-3 could be detected, in accordance with the findings observed at gene level. Moreover IL-6, IL-8, and TGF-beta 1 concentrations were high at different time points. EGF, analyzed as a negative control since the expression of the coding gene was unchanged, was not increased in the supernatant. In the supernatant of fibroblasts, we detected a very high concentration of MCP-1. Also, MCP-3 was elevated at all time points, and IP10 was released at high levels at late time points. We found increased levels of the interleukins IL-6, IL-8, and IL-11, reflecting the upregulation of the corresponding genes. Both TGF-beta 1 and VEGF concentrations were increased, and CTGF was detectable by Western blot. Finally, Collagen type I was secreted at all time points, indicating that fibroblasts were functionally active and were secreting constituents of the extracellular matrix, a finding that is compatible with the phenotype of scleroderma fibroblasts. These data show that gene overexpression is paralleled by increased secretion of the corresponding molecules in the supernatant of stimulated cells.

**Table 6:**
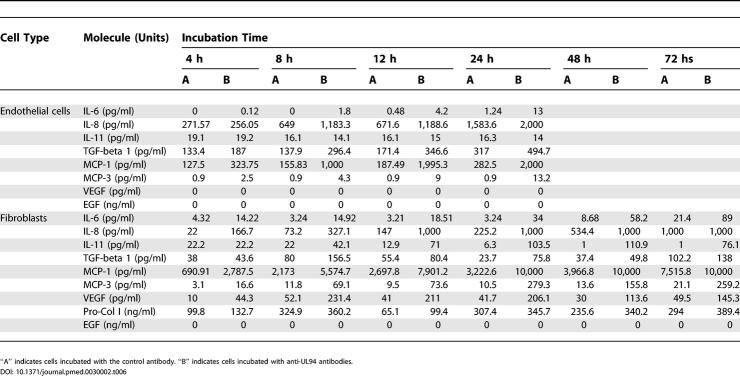
Soluble Mediators Released in Cell Supernatants

### Analysis of Soluble Molecule Concentrations in the Sera of SSc Patients

To investigate whether our findings from the gene array analysis, Q-PCR, and biochemical analysis of the cell supernatants have a functional correspondance in vivo, we analyzed the sera of 81 patients affected by SSc. The levels of IL-6, IL-8, IL-11, ET-1, MCP-1, MCP-3, soluble ICAM-1, soluble VCAM-1, and soluble E-selectin were detected by ELISA (Table 7). The levels of IL-6, IL-8, ET-1, soluble ICAM-1, soluble VCAM-1, VEGF, and soluble E-selectin were significantly higher in patients with SSc than in sex- and age-matched control individuals. When the limited and the diffuse forms of the disease were considered, ET-1, MCP-1, soluble ICAM-1, and soluble E-selectin were significantly higher in patients with diffuse SSc, whereas soluble VCAM-1 was higher in patients with the limited form of the disease (Table 8). MCP-3 and IL-11 levels were not different in patients and controls. These data confirmed the in vitro findings in vivo for the majority of the molecules analyzed.

**Table 7:**
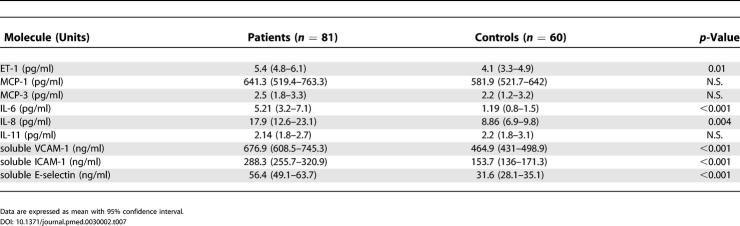
Soluble Mediator Levels in the Sera of Patients and Controls

**Table 8:**
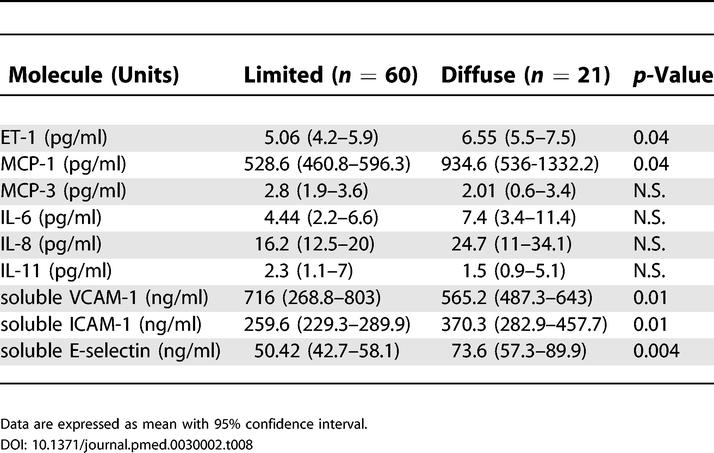
Soluble Mediator Levels in the Sera of Patients Affected by Limited and Diffuse SSc

### Functional Analysis of Gene Expression in Endothelial Cells and Fibroblasts

GO analysis was used to organize the gene expression data into their functional relationships based on molecular-level and biologic processes, and revealed several GO groups that are of key interest in SSc endothelial cell and fibroblast pathophysiology. For endothelial cells some of the most representative groups included cell adhesion, integrin-mediated signaling pathway, and apoptosis, and for fibroblasts the groups included development and cell proliferation, cell adhesion, immune response, extracellular matrix organization and biosynthesis, and muscle development (Tables S1–S4).

## Discussion

In the present report we demonstrate that the purified subset of antibodies directed against the hCMV-derived protein UL94 bind dermal fibroblasts through the surface receptor NAG-2. Following this interaction, fibroblasts do not undergo apoptosis, as endothelial cells do, but proliferate normally and acquire an activated phenotype resembling the features of “scleroderma-like” fibroblasts. Therefore, this subset of anti-hCMV antibodies seems to promote not only endothelial cell apoptosis, but also fibroblast activation upon engagement of the same receptor molecule, NAG-2. To further investigate the effects induced in these two different cellular targets by the anti-UL94 antibody population, we used a gene array approach, which allows the simultaneous detection of thousands of genes in a given sample. By this approach we found that the purified anti-hCMV antibodies modulate a vast array of genes encoding molecules that play a pivotal role in the pathogenesis of SSc.

In endothelial cells some of these genes encode molecules that are induced under the influence of proinflammatory cytokines, such as Tumor necrosis factor alpha (TNF-alpha) and IL-1 [45]. Indeed, genes encoding adhesion molecules such as ICAM-1, VCAM-1, E-selectin, and P-selectin were upregulated, and the findings were confirmed by Q-PCR. These molecules show increased expression on endothelial cells of SSc skin lesions, and the corresponding soluble forms are increased in the serum of SSc patients [20,46]. Moreover, ICAM-1 seems to correlate with the severity of the disease [47] and E-selectin with the presence of pulmonary fibrosis [48]. We detected a significant increase of these adhesion molecules both in the supernatant of cultured cells and in the sera of the patients analyzed.

Significant abnormalities in chemokine expression have been found in SSc [22,49,50], and, indeed, different chemokine-encoding genes were activated in treated endothelial cells. The gene encoding MCP-1 presented the highest rate of activation among all the genes considered. This result was confirmed by the high increase in MCP-1 transcripts shown by Q-PCR. MCP-1 plays a pivotal role in the pathogenesis of SSc, and, indeed, it is expressed at high levels by inflammatory mononuclear cells and endothelial cells in the skin of patients with SSc of recent onset, suggesting that this molecule may be involved in the first stages of the disease [49]. Moreover, elevated levels of MCP-1 are present in bronchoalveolar lavage cells from SSc patients with lung involvement, and high serum levels of MCP-1 correlate with pulmonary fibrosis, suggesting a potential role of this chemokine in the pathogenesis of lung damage [49]. Also the gene encoding MCP-3 was upregulated, and, indeed, this molecule has been found to be elevated in early-stage SSc and may act as a fibrotic mediator activating extracellular matrix gene expression in addition to promoting leukocyte trafficking [50]. A similar behavior has been observed for MIP-1 alpha [49,51] and GRO alpha [52]. The chemokine SDF-1 was also upregulated; since hypoxia is a potent stimulus for SDF-1, we can hypothesize that during early vascular damage the hypoxic microenvironment may induce the release of SDF-1, as demonstrated in another autoimmune disease, rheumatoid arthritis [53]. And lastly among chemokines, Fractalkine was highly induced in endothelial cells exposed to anti-hCMV antibodies (with a fold increase in expression of 4.5 at 4 h of treatment); this chemokine has been found to be overexpressed in endothelial cells of affected skin and in the lung tissues of patients with SSc. Soluble fractalkine levels were significantly raised in sera of patients with SSc and were associated with digital ischemia and severity of pulmonary fibrosis [54].

Among the other genes found activated, of particular relevance are the genes that encode ET-1 and PDGF. ET-1 is a potent vasoconstrictor molecule known to be associated with vascular damage in cardiovascular disease [55]. ET-1 is also able to induce fibrosis by enhancing collagen synthesis and inhibiting expression of MMP-1 in skin fibroblasts. Indeed, ET-1 induces a fibrogenic phenotype in normal fibroblasts that is similar to that of lesional SSc fibroblasts [56,57]. ET-1 has been extensively studied in patients with SSc who present high circulating levels of the molecule [58]. The profibrotic cytokine PDGF plays a major role in stimulating the replication, survival, and migration of fibroblasts during the pathogenesis of fibrotic diseases. Moreover, it seems to be able to induce a significant increase in MCP-1 messenger RNA and protein in cultured fibroblasts [59]. Finally, the upregulation of apoptosis-related genes is in accordance with the reported endothelial cell apoptosis induced by anti-UL94 antibodies.

In fibroblasts, the genes upregulated are mainly involved in cell activation and proliferation and in the production of extracellular matrix, all features of scleroderma. In particular, proinflammatory and profibrotic cytokines such as IL-6 and IL-8 may act synergistically with the chemokines MCP-1 and MCP-3 in recruiting monocytes and leukocytes at the site of inflammation and acting as fibrotic mediators. The upregulation of genes encoding IL-6, IL-8, and MCP-1 have been confirmed by the high increase in the corresponding transcripts detected by Q-PCR.

MCP-1 and MCP-3 have been shown to be overexpressed in SSc fibroblasts [22,50]. The production of MCP-1 by fibroblasts may be mediated by PDGF [59], whose gene is upregulated in treated endothelial cells and fibroblasts. Moreover, the gene encoding PDGF receptor B was overexpressed in fibroblasts treated with anti-hCMV antibodies, and it is known that PDGF action is determined by the relative expression of PDGF receptors on the surface of myofibroblasts. These receptors are induced during fibrogenesis, thereby amplifying the biological responses to PDGF.

The gene encoding the profibrotic cytokine TGF-beta 1 was also overexpressed. TGF-beta 1 represents a major stimulator of collagen gene expression through two different signaling pathways, immediate and delayed [26]. The first one involves proteins known as Smads that are phosphorylated by activated TGF-beta receptors, leading to nuclear translocation and gene transactivation. The second one involves the induction of secondary matrix stimulatory proteins by TGF-beta 1, amplifying the initial signal. This activation pathway involves CTGF [26]. Both Smad- and CTGF-encoding genes were modulated by the anti-hCMV antibodies in dermal fibroblasts. TGF-beta 1 may also induce apoptosis resistance of SSc skin fibroblasts by activating Akt, a kinase of the phosphoinositide-3-kinase/Akt signaling pathway with potent anti-apoptotic effects [36]; the resistance to apoptosis of SSc fibroblasts probably contributes to the accumulation of activated fibroblasts in the skin, promoting the deposition of extracellular matrix. Moreover, Akt has been implicated in cellular transdifferentiation; since TGF-beta 1 has been implicated in the conversion of fibroblasts to myofibroblasts, Akt activation may play a role in the increased number of myofibroblasts that is a key feature of SSc [36]. Moreover, the phosphoinositide-3-kinase/Akt pathway seems to be implicated in endothelial cell induced smooth muscle cell differentiation [60] also through ET-1 signaling [61]. Finally, EGF has been recently reported to induce upregulation of TGF-beta receptor through the phosphoinositide-3-kinase/Akt signaling pathway [62]. In accordance with characteristics of SSc, we found upregulation of the genes encoding Akt and EGF receptor, and of genes typically expressed by smooth muscle cells, in the fibroblasts exposed to anti-hCMV antibodies.

Chronic uncontrolled VEGF upregulation seems responsible for the disturbed vessel morphology in the skin of patients with SSc, and the high serum VEGF levels may be an indicator of capillary damage in SSc [63,64]. We found both overexpression of the gene encoding VEGF in cultured fibroblasts and high circulating levels of VEGF in our patients. Of particular relevance also is the upregulation of Angiotensin II receptor type 1 in endothelial cells and in fibroblasts; this receptor plays a pivotal role in ischemia-induced angiogenesis and in tissue fibrosis through excessive production of extracellular matrix components [24,35].

We also tested the level of some chemokines, cytokines, growth factors, and Collagen type I in the supernatants of stimulated and unstimulated cells and found that the concentration of the molecules measured was increased in the cells incubated with anti-hCMV antibodies, confirming that gene upregulation is paralleled by the induction of protein synthesis.

Finally, we measured the serum concentrations of some cytokines, chemokines, and adhesion molecules in patients and controls in order to confirm that the genes found overexpressed in vitro following stimulation with anti-hCMV antibodies could indeed be of relevance in vivo. We found that the levels of the majority of these molecules were significantly higher in patients than in controls, with a difference between the diffuse and limited subsets of the disease for some molecules, including MCP-1 and ET-1. A number of these soluble markers have already been reported to be increased in the serum of SSc patients [20]. The normal serum concentration of some other molecules, such as MCP-3, may be related to the presence of an increased level only in the damaged tissue, e.g., in the lungs of patients with lung fibrosis.

In conclusion, our results further support the pathogenic role of antibodies against the hCMV late protein UL94 in SSc. We found these antibodies in the vast majority of Caucasian patients with SSc from northern Italy [11]; the same antibodies have been detected in both Caucasian and African American patients, and their concentrations have been associated with the severity of the disease [65]. We show here that these anti-virus antibodies are able to induce not only endothelial cell activation and apoptosis but also fibroblast activation. They would therefore act as a unifying stimulus that may explain vascular damage and fibrosis, the two hallmarks of SSc.

## Supporting Information

Dataset S1 Genes Upregulated in Endothelial Cells at 4 and 8 h of Incubation with Anti-hCMV Affinity Purified Antibodies (689 KB XLS).

Dataset S2 Genes Upregulated in Human Fibroblasts at 4 and 8 h of Incubation with Anti-hCMV Affinity Purified Antibodies (380 KB XLS).

Dataset S3 Genes Downregulated in Endothelial Cells at 4 and 8 h of Incubation with Anti-hCMV Affinity Purified Antibodies (525 KB XLS).

Dataset S4 Genes Downregulated in Human Fibroblasts at 4 and 8 h of Incubation with Anti-hCMV Affinity Purified Antibodies (371 KB XLS).

Table S1 GO Listing of Upregulated Genes in HUVEC Cells (36 KB XLS).

Table S2 GO Listing of the Upregulated Genes in Fibroblasts (36 KB XLS).

Table S3 GO Listing of Downregulated Genes in Endothelial Cells (29 KB XLS).

Table S4 GO Listing of Downregulated Genes in Fibroblasts (39 KB XLS).

**Patient Summary**

**Background**

Systemic sclerosis, or scleroderma, is the name of a progressive disease that is characterized by the abnormal growth of connective tissue and by the narrowing of small blood vessels. It is triggered when the body's immune system turns against the body, causing abnormal production of collagen, which can be limited to the skin or extend to internal organs. Two types of cells that are involved in systemic sclerosis are the endothelial cells that line the blood vessels and fibroblasts, which are involved in the increased fibrosis seen in the skin in systemic sclerosis.

**Why Was This Study Done?**

Previously these researchers have shown that one trigger for systemic sclerosis might be antibodies to a protein from a common human virus, cytomegalovirus, which also reacts with a molecule on the surface of endothelial cells and causes them to die. The researchers wanted to look more at this possible mechanism of disease and work out exactly how these antibodies affected endothelial cells and fibroblasts.

**What Did the Researchers Do and Find?**

They looked first in cells cultured in the laboratory to see if the antibody that they had found previously also stuck to fibroblasts. They found that it did, and that the attachment of this antibody caused a change in expression of many genes in both fibroblasts and endothelial cells. For example, in fibroblasts there was an increased expression of several genes that code for collagen—which is increased in fibrosis.

**What Do These Findings Mean?**

These findings suggest that an antibody to a common virus can trigger changes in cells similar to those seen in systemic sclerosis. Although there are no immediate implications for treatment, these results may help researchers to understand more about why systemic sclerosis develops.

**Where Can I Get More Information Online?**

MedlinePlus has many links to pages of information on systemic sclerosis:

http://www.nlm.nih.gov/medlineplus/scleroderma.html

The Scleroderma Foundation is a nonprofit organization based in the United States that provides information on scleroderma for patients, and supports research:

http://www.scleroderma.org/

## Abbreviations

CSF: colony-stimulating factor
ELISA: enzyme-linked immunosorbent assay
F.C.: fold change
GO: Gene Ontology
hCMV: human cytomegalovirus
HUVEC: human endothelial cell
Q-PCR: quantitative real-time polymerase chain reaction
SSc: systemic sclerosis
